# Supplementary material for: Trophic niche overlap between coyotes and gray foxes in a temperate forest in Durango, Mexico
Source: PLoS One. 2021 Dec 1;16(12):e0260325. doi: 10.1371/journal.pone.0260325 (PMC8635360; doi:10.1371/journal.pone.0260325)
Supplement: S1 File — (DOCX) [file pone.0260325.s002.docx]

**Resumen**

El reparto de recursos, especialmente los alimentarios, es un mecanismo que se ha estudiado para varias especies de cánidos como un medio para comprender las relaciones competitivas y la capacidad para coexistir de estas especies. En México, el coyote (*Canis latrans*) y la zorra gris (*Urocyon cinereoargenteus*) son dos especies de cánidos que se encuentran ampliamente distribuidos y son simpátricos en la mayor parte de su rango de distribución geográfica. Sin embargo, la dinámica trófica y la superposición entre sus dietas no ha sido investigada a fondo. Con el objetivo de comprender mejor sus relaciones ecológicas e interacciones competitivas, investigamos la superposición de nicho trófico entre ambos cánidos en un bosque templado de Durango, México. Los resultados se basaron en el análisis de 540 excrementos de coyote y 307 de zorra gris, colectadas en el año 2018. Ambas especies consumieron una gama similar de alimentos, pero el coyote consumió especies de talla grande que la zorra gris no incorporó en su dieta. Para ambas especies, las categorías tróficas con mayor frecuencia de consumo durante el año y estacionalmente fueron los frutos y los mamíferos silvestres (principalmente roedores y lagomorfos). Los coyotes mostraron mayor diversidad trófica en su dieta anual (*H´* = 2.33) que las zorras grises (*H´* = 1.80). Al analizar las dietas por temporada, la diversidad trófica de ambas especies fue mayor en invierno y primavera y tendió a disminuir en verano y otoño. Al comparar entre especies, este parámetro difirió significativamente durante todas las estaciones excepto en verano. La superposición trófica anual fue alta (*R_0_* = 0.934), con una variación estacional entre *R_0_* = 0.821 (otoño) y *R_0_* = 0.945 (primavera). Ambas especies basaron su dieta en los alimentos de mayor disponibilidad durante cada estación del año, mostrando alta superposición trófica, que probablemente conduce a intensos procesos de competencia de explotación. Sin embargo, la diferencia en la diversidad trófica causada por el uso diferencial de presas demostró ser uno de los medios que permite que estas especies de cánidos de diferentes tamaños corporales coexistan en el área de estudio.
