# Supplementary material for: Trophic niche overlap between coyotes and gray foxes in a temperate forest in Durango, Mexico
Source: PLoS One. 2021 Dec 1;16(12):e0260325. doi: 10.1371/journal.pone.0260325 (PMC8635360; doi:10.1371/journal.pone.0260325)

PLOS ONE

Trophic niche overlap between coyotes and gray foxes in a temperate forest in Durango, Mexico

--Manuscript Draft--

**Manuscript Number:**	PONE-D-21-19581
**Article Type:**	Research Article
**Full Title:**	Trophic niche overlap between coyotes and gray foxes in a temperate forest in Durango, Mexico
**Short Title:**	Trophic overlap between coyotes and gray foxes
**Corresponding Author:**	David Valenzuela-Galván<br>Centro de Investigacion en Biodiversidad y Conservacion, Universidad Autónoma del Estado de Morelos<br>Cuernavaca, Morelos MEXICO
**Keywords:**	Canidae, Canis latrans, Urocyon cinereoargenteus, Food habits, competition, coexistence, Natural Protected Area
**Abstract:**	Resource partitioning, especially dietary, is a mechanism that has been studied for several canid species as a means to understand competitive relationships and the ability of these species to coexist. In México, coyotes (Canis latrans) and gray foxes (Urocyon cinereoargenteus) are two canid species that are widely distributed, and they are sympatric throughout most of their distribution range. However, trophic dynamic and overlap between them has not been thoroughly studied. In order to better understand their ecological relationship and competitive interactions, we studied the trophic niche overlap between both canids in a temperate forest of Durango, Mexico. The results are based on the analysis of 540 coyote and 307 gray fox feces collected in 2018. Both species consumed a similar range of food items, but the coyote consumed large species while the gray fox did not. For both species, the most frequently consumed food categories throughout the year and seasonally were fruit and wild mammals (mainly rodents and lagomorphs). Coyotes had higher trophic diversity in their annual diet (H' = 2.33) than gray foxes (H' = 1.80). When analyzing diets by season, trophic diversity of both species was higher in winter and spring and tended to decrease in summer and autumn. When comparing between species, this parameter differed significantly during all seasons except for summer. Trophic overlap throughout the year was high (R 0 = 0.934), with seasonal variation between R 0 = 0.821 (autumn) and R 0 = 0.945 (spring). Both species based their diet on the most available food items throughout each season of the year, having high dietary overlap which likely leads to intense exploitative competition processes. However, difference in trophic diversity caused by differential prey use proved to be one of the means that allows these different body sizes canid species to coexist in the study area.
**Order of Authors:**	César Ricardo Rodríguez-Luna
	Jorge Servín
	David Valenzuela-Galván
	Rurik List
**Additional Information:**	
**Question**	**Response**
**Financial Disclosure**<br><br>Enter a financial disclosure statement that describes the sources of funding for the work included in this submission. Review the submission guidelines for detailed requirements. View published research	CRRL received funding from CONACYT through the doctoral grant #293353; CONACYT as funder, had no role in study design, data collection and analysis, decision to publish, or preparation of the manuscript.

Powered by Editorial Manager® and ProduXion Manager® from Aries Systems Corporation

articles from *PLOS ONE* for specific examples.<br><br>This statement is required for submission and **will appear in the published article** if the submission is accepted. Please make sure it is accurate.<br><br>**Unfunded studies**<br>Enter: *The author(s) received no specific funding for this work.*<br><br>**Funded studies**<br>Enter a statement with the following details:<br>• Initials of the authors who received each award<br>• Grant numbers awarded to each author<br>• The full name of each funder<br>• URL of each funder website<br>• Did the sponsors or funders play any role in the study design, data collection and analysis, decision to publish, or preparation of the manuscript?<br>• **NO** - Include this sentence at the end of your statement: *The funders had no role in study design, data collection and analysis, decision to publish, or preparation of the manuscript.*<br>• **YES** - Specify the role(s) played.<br><br>* typeset	
**Competing Interests**<br><br>Use the instructions below to enter a competing interest statement for this submission. On behalf of all authors, disclose any competing interests that could be perceived to bias this work—acknowledging all financial support and any other relevant financial or non-financial competing interests.<br><br>This statement is required for submission and **will appear in the published article** if the submission is accepted. Please make sure it is accurate and that any funding sources listed in your Funding Information later in the submission form are also declared in your Financial Disclosure statement.	The authors have declared that no competing interests exist

View published research articles from *PLOS ONE* for specific examples.<br><br>**NO authors have competing interests**<br><br>Enter: *The authors have declared that no competing interests exist.*<br><br>**Authors with competing interests**<br><br>Enter competing interest details beginning with this statement:<br><br>*I have read the journal's policy and the authors of this manuscript have the following competing interests: [insert competing interests here]*<br><br>* typeset	
**Ethics Statement**<br><br>Enter an ethics statement for this submission. This statement is required if the study involved:<br><br>• Human participants<br>• Human specimens or tissue<br>• Vertebrate animals or cephalopods<br>• Vertebrate embryos or tissues<br>• Field research<br><br>Write "N/A" if the submission does not require an ethics statement.<br><br>General guidance is provided below. Consult the submission guidelines for detailed instructions. **Make sure that all information entered here is included in the Methods section of the manuscript.**	Field research and scat collection was supported by Collection Permit extended to JS by the Dirección General de Vida Silvestre (SEMARNAT; SGPA/DGVS/12685/18)

Powered by Editorial Manager® and ProduXion Manager® from Aries Systems Corporation

**Format for specific study types**<br><br>**Human Subject Research (involving human participants and/or tissue)**<br>• Give the name of the institutional review board or ethics committee that approved the study<br>• Include the approval number and/or a statement indicating approval of this research<br>• Indicate the form of consent obtained (written/oral) or the reason that consent was not obtained (e.g. the data were analyzed anonymously)<br><br>**Animal Research (involving vertebrate animals, embryos or tissues)**<br>• Provide the name of the Institutional Animal Care and Use Committee (IACUC) or other relevant ethics board that reviewed the study protocol, and indicate whether they approved this research or granted a formal waiver of ethical approval<br>• Include an approval number if one was obtained<br>• If the study involved *non-human primates*, add *additional details* about animal welfare and steps taken to ameliorate suffering<br>• If anesthesia, euthanasia, or any kind of animal sacrifice is part of the study, include briefly which substances and/or methods were applied<br><br>**Field Research**<br><br>Include the following details if this study involves the collection of plant, animal, or other materials from a natural setting:<br>• Field permit number<br>• Name of the institution or relevant body that granted permission	
**Data Availability**<br><br>Authors are required to make all data underlying the findings described fully available, without restriction, and from the time of publication. PLOS allows rare exceptions to address legal and ethical concerns. See the PLOS Data Policy and FAQ for detailed information.	No - some restrictions will apply

A Data Availability Statement describing where the data can be found is required at submission. Your answers to this question constitute the Data Availability Statement and **will be published in the article**, if accepted.<br><br>**Important:** Stating 'data available on request from the author' is not sufficient. If your data are only available upon request, select 'No' for the first question and explain your exceptional situation in the text box.<br><br>Do the authors confirm that all data underlying the findings described in their manuscript are fully available without restriction?	
**Describe where the data may be found in full sentences. If you are copying our sample text, replace any instances of XXX with the appropriate details.**<br><br>• If the data are **held or will be held in a public repository**, include URLs, accession numbers or DOIs. If this information will only be available after acceptance, indicate this by ticking the box below. For example: *All XXX files are available from the XXX database (accession number(s) XXX, XXX.*).<br>• If the data are all contained **within the manuscript and/or Supporting Information files**, enter the following: *All relevant data are within the manuscript and its Supporting Information files.*<br>• If neither of these applies but you are able to provide **details of access elsewhere**, with or without limitations, please do so. For example:<br><br>*Data cannot be shared publicly because of [XXX]. Data are available from the XXX Institutional Data Access / Ethics Committee (contact via XXX) for researchers who meet the criteria for access to confidential data.*<br><br>*The data underlying the results presented in the study are available from (include the name of the third party*	All relevant data are within the manuscript and its Supporting Information files.

and contact information or URL).<br>• This text is appropriate if the data are owned by a third party and authors do not have permission to share the data.<br><br>* typeset	
Additional data availability information:	

Manuscript Click here to access/download;Manuscript;Trophic overlap coyotes gray foxes_RodriguezLuna et al POne.docx

Trophic niche overlap between coyotes and gray foxes in a temperate forest in Durango, Mexico

Trophic overlap between coyotes and gray foxes

César Ricardo Rodríguez-Luna[1], Jorge Servín[2], David Valenzuela-Galván[3]*, and Rurik List[4]

[1]Doctorado en Ciencias Biológicas y de la Salud, Universidad Autónoma Metropolitana, Mexico.

[2]Departamento El Hombre y su Ambiente, División de Ciencias Biológicas y de la Salud, Universidad Autónoma Metropolita–Unidad Xochimilco, Coyoacán, Mexico City, Mexico.

[3]Departamento de Ecología Evolutiva, Centro de Investigación en Biodiversidad y Conservación, Universidad Autónoma del Estado de Morelos, Cuernavaca, Morelos, Mexico.

[4]Departamento de Ciencias Ambientales, División de Ciencias Biológicas y de la Salud, Universidad Autónoma Metropolitana–Unidad Lerma, Lerma de Villada, Mexico, Mexico.

*Corresponding author

E-mail: dvalen@uaem.mx (DVG)

Abstract

Resource partitioning, especially dietary, is a mechanism that has been studied for several canid species as a means to understand competitive relationships and the ability of these species to coexist. In México, coyotes (*Canis latrans*) and gray foxes (*Urocyon cinereoargenteus*) are two canid species that are widely distributed, and they are sympatric throughout most of their distribution range. However, trophic dynamic and overlap between them has not been thoroughly studied. In order to better understand their ecological relationship and competitive interactions, we studied the trophic niche overlap between both canids in a temperate forest of Durango, Mexico. The results are based on the analysis of 540 coyote and 307 gray fox feces collected in 2018. Both species consumed a similar range of food items, but the coyote consumed large species while the gray fox did not. For both species, the most frequently consumed food categories throughout the year and seasonally were fruit and wild mammals (mainly rodents and lagomorphs). Coyotes had higher trophic diversity in their annual diet (H' = 2.33) than gray foxes (H' = 1.80). When analyzing diets by season, trophic diversity of both species was higher in winter and spring and tended to decrease in summer and autumn. When comparing between species, this parameter differed significantly during all seasons except for summer. Trophic overlap throughout the year was high (R_0 = 0.934), with seasonal variation between R_0 = 0.821 (autumn) and R_0 = 0.945 (spring). Both species based their diet on the most available food items throughout each season of the year, having high dietary overlap which likely leads to intense exploitative competition processes. However, difference in trophic diversity caused by differential prey use proved to be one of the means that allows these different body sizes canid species to coexist in the study area.

Introduction

The trophic dimension of species' ecological niches is important because it can determine the structure of ecological communities [1] due to the importance of food resources for animals. Therefore, understanding the ways that species partition this resource contributes to the understanding of interactions among sympatric species [2]–[3]. When species occur in sympatry, the competitive exclusion principle [4] proposes that the species segregate their ecological niches in at least one of their dimensions in order to reduce interspecific competition [5]–[7]. One of the most important forms of resource partitioning in ecological communities is differentiation of the use of food resources [8], in such a way that extensive trophic overlap between ecologically similar species is relatively rare [9]. Comparing the food habits of sympatric species reveals the overlap degree in their trophic niche, which can be interpreted as a measure of the potential for interspecific competition between them [10]–[11], and thus provides ~~an idea~~ of the mechanisms that reduce their competitive interactions in order to maintain sympatry [12]. Although there is abundant evidence of this kind of complex interactions among carnivorous mammals, these interactions are poorly understood for many species [13].

Coyotes (*Canis latrans*) and gray foxes (*Urocyon cinereoargenteus*) are sympatric over a large part of their range in North America [14]–[15]; however, the ecological relations between them have received little attention [10]. These two canids are generalist-opportunist species that consume similar food items [14]–[15] and they potentially compete for similar resources. North of its distribution the overlap of its diets can vary between medium [16]–[17] and high proportions [10], [18]–[19]. But in the southern portion of its distribution range, trophic dynamic and dietary overlap between coyotes and gray foxes has not been thoroughly studied. In a

tropical dry forest in southern Mexico, it was reported that they showed intermediate trophic niche overlap and low potential for interspecific competition between them [20]; while in the north of the country, in a temperate forest, they showed intermediate–high dietary overlap proportion [21]. As trophic level mechanisms that allow the coexistence of these two canids in the country remain unclear, a study of food resource partitioning in sympatric coyote and gray fox populations in Mexico will help to understand their competitive relations and to elucidate a possible mechanism for stable coexistence between these two canids, through trophic niche segregation. Our objective was thus to analyze the trophic interactions and evaluate the potential for interspecific competition for food resources between coyotes and gray foxes in a temperate forest of the Sierra Madre Occidental, in a protected reserve, in the state of Durango, Mexico.

We analyzed the indigestible contents of feces of both species to determine their diet composition, the relative importance of different food items, and its seasonal variation. Using those data, we evaluated: (1) the trophic diversity of these two species, (2) whether this variable differed between the species, and (3) the similarity of diets as a measure of trophic niche overlap, considering a high overlap degree as an indicator of high potential for exploitative competition [12], [22]. We expected significant differences in trophic diversity between the species, and therefore, low trophic niche overlap, since the coyote (up to 16 kg in the study area [23]) is a larger species than the gray fox (3–5 kg [24]) and should thus consume a wider range of food resources, increasing its trophic niche breadth, compared to the gray fox, as has been reported for this species in other parts of its geographic distribution range.

Materials and Methods

Study area

We carried out this work in the buffer zone of “La Michilía” Biosphere Reserve (MBR), in the municipality of Súchil, Durango, Mexico, located between the coordinates 23° 21’–23° 28’ N and 104° 09’–104° 21’ W (Fig 1). The MBR is found in the transition zone between the Neartic and Neotropical biogeographic zones [25]–[26]. The MBR is bordered by the Sierra de Urica to the west, which is gently sloped, and by the Sierra de Michis on the east, which has steep depressions and marked slopes [27]. The altitude of the study zone varies between 2,000–2,985 m [28]. In the northern part of the MBR the climate is semi-dry temperate (BS1k) and in the rest of the MBR the dominant climate is temperate sub-humid (Cw [29]). Mean monthly temperatures range from 2°C in February to 22°C in July and the average annual precipitation ranged from 600–900 mm [30].

Fig 1. Geographic location of the study area in the buffer zone of La Michilía Biosphere Reserve (MBR), Durango, Mexico.

Dominant vegetation within the MBR is coniferous (*Pinus* spp.) and oak (*Quercus* spp.) forest, though there are also zones of natural grassland (*Bouteloua* spp.), xerophytic scrub (*Arctostaphylos pungens*, *Acacia schaffneri*), and aquatic vegetation; in addition, the MBR has transition zones among these vegetation types, leading to the formation of mixed forests [31].

Sample collection and identification

Each month during 2018, we collected feces of coyotes and gray foxes along paths, main and secondary roads, and streams, in all the vegetation types present in the study area. Prior to the study, we removed all feces from the study area, allowing us to date the samples to the nearest month during the study period. We also disregarded feces that could not be reliably

identified. We identified feces to the species level by size, shape, and color of each sample, and we considered as complementary evidence the presence of tracks of the focal species at the site at the time of collection [32]–[34]. Subsequently, to reduce assignment error, we used feces diameter as a criterion to identify canid species in the study area, see [35]. Thus, we assigned to *C. latrans* all feces that had maximum diameter between 18.10–33.00 mm, and to *U. cinereoargenteus* the feces with maximum diameter between 9.00–16.9 mm [35], so we excluded from data analysis feces with maximum diameter between 17.00–18.00 mm, as well as all disintegrated samples, accounting for 7.43% (n = 68) of the total feces we collected.

We categorized the feces into four seasons based on food-availability cycles [35]: winter (December 22–March 20), spring (March 21–June 20), summer (June 21–September 20), and autumn (September 21–December 21). We placed the feces individually in paper bags, labeled them with the species name, date, season, and geographic location, to be air dried. In laboratory, we transferred all feces to nylon stockings and washed with water and commercial detergent to remove soluble material. We then manually disintegrated the feces and dried them in a 65°C oven for 48 h [35]. After drying, we recovered all the undigested parts of food items (hairs, scales, exoskeletons, bones, skin, teeth, feathers, and seeds); in the case of hairs, we mounted samples of them on microscope slides to visualize their medulla and scale patterns [36]. We then identified the undigested fragments to the lowest taxonomic level possible using reference samples from the study area from the collection of the Wildlife Ecology and Conservation Laboratory (*Laboratorio de Ecología y Conservación de Fauna Silvestre*) of the Universidad Autónoma Metropolitana–Unidad Xochimilco, as well as specialized literature [37]–[41]. We classified the undigested fragments (i. e. food items) from both canids into six trophic categories: 1) fruits, 2) invertebrates, 3) reptiles, 4) birds, 5) livestock (caprine, ovine, and bovine), and 6)

wild mammals. In the case of herbs and/or grass (n = 4), we assumed that they were incidentally ingested while consuming small prey or to aid in the digestive process [10], so we excluded them from subsequent analyses.

Sample analysis

For each species, the overall and seasonally representation of each food item and food category was expressed as: 1) number of occurrences (n), n_i = number of feces containing prey item i; and 2) frequency of occurrence (FO), FO_i (%) = (n_i / N) * 100, where N is the total number of feces [18], [42]. The FO measures the percentage of feces that contain a given prey item, and although do not necessarily approximate the volumetric importance of items in the diet, it indicates the relative importance role of items in the diet [21], [43], how common an item was in the diet [44], and can provide valuable insight into carnivore ecology [18], [45].

We used Clench's asymptotic species accumulation model to estimate the completeness of the sampling. To do this, the data of food items found in feces were randomized 1000 times with the program *EstimateS* version 9.1.0 [46]. Additionally, we used Fisher's exact test [47] to analyze whether the distribution of prey items among the trophic categories varied between species and between seasons. We also calculated Shannon's diversity index (H' [48]) to estimate the trophic diversity, and to identify possible significant differences in trophic diversity between species and among seasons using a Hutcheson's t test [49], with the program Past 4.03 [50]. To estimate the trophic niche overlap we used Horn's index (R_0 [51]), corrected to avoid bias due to undersampling [52]. The index values range from 0 (no overlap) to 1 (complete overlap). For most of the statistical analysis we used R version 4.0.2 program [53].

Results

We analyzed a total of 847 feces, 540 from coyotes and 307 from gray foxes. We identified 25 different food items for the coyote and 17 for the gray fox. The Clench model predicted 27.16 and 18.76 food items according to the models for coyote ($R^2 = 0.998$) and gray fox ($R^2 = 0.978$), respectively. Thus, in the case of coyotes it reached 92.05% and in the case of gray foxes 90.62% of the total food items expected.

Overall diet composition

Fisher's exact test indicated that prey items distribution across trophic categories differed significantly ($P < 0.001$) between coyote and gray fox feces. In the case of coyotes, we identified 25 different food items from all six trophic categories. The category with the highest frequency of occurrence value was fruits ($FO_f = 68.52$), followed by wild mammals ($FO_{wm} = 47.59\%$) and invertebrates ($FO_i = 6.48$; Table 1). The fruits category was represented by cedar fruits (*Juniperus deppeana*), which was the predominant food item in coyote feces ($FO_{Jd} = 39.07$), followed by fruits of pointleaf manzanita (*A. pungens*; $FO_{Ap} = 29.44$). Among wild mammals category, rodents were the most frequent food item, represented by seven species; the most frequently consumed were mice of the genus *Peromyscus* ($FO_P = 10.74$) and *Sigmodon* ($FO_S = 8.52$). In the same category, the next most frequent food items were from the orders Lagomorpha, represented by *Sylvilagus audubonii* and *Lepus californicus* from Leporidae family ($FO_L = 8.52$); and Artiodactyla, represented by *Odocoileus virginianus* ($FO_{Ov} = 3.15$), *Pecari tajacu* ($FO_{Pt} = 2.04$) and the exotic species *Sus scrofa* ($FO_{Ss} = 1.67$). Among invertebrates, the most frequent food item was of the order Coleoptera ($FO_C = 5.00$), while, among birds, was

Meleagris gallopavo (FO_{Mg} = 2.41). In the livestock category, we found traces of cattle (*Bos taurus*), goats (*Capra hircus*), and sheep (*Ovis aries*), all with *FO* values ≤ 1.11 (Table 1).

Table 1. Total number of samples (*N*) and trophic diversity (*H'*) of the coyote and gray fox, as well as overall number of occurrences (n_i) and frequency of occurrence (*FO%*) of food items by trophic category, in both canids diets, in the buffer zone of La Michilia Biosphere Reserve (MBR), Durango, Mexico.

	**Coyote**		**Gray fox**	
	***N* = 540**		***N* = 307**	
	***H'* = 2.33**		***H'* = 1.80**	
**Food item and trophic category**	n_i	***FO%***	n_i	***FO%***
**Wild mammals category**	**257**	**47.59**	**88**	**28.66**
CARNIVORA				
Mephitidae	12	2.22	1	0.33
Procyonidae				
Nasua narica	1	0.19	2	0.65
Procyon lotor	1	0.19	0	0
ARTIODACTYLA				
Cervidae				
Odocoileus virginianus	17	3.15	0	0
Tayassuidae				
Pecari tajacu	11	2.04	0	0
Suidae				
Sus scrofa	9	1.67	0	0
RODENTIA				
Sciuridae				
Sciurus nayaritensis	10	1.85	3	0.98
Otospermophilus variegatus	7	1.30	2	0.65
Geomyidae				
Thomomys umbrinus	7	1.30	3	0.98
Heteromyidae				
Heteromys irroratus	5	0.93	0	0
Cricetidae				
Peromyscus sp.	58	10.74	36	11.73
Reithrodontomys sp.	1	0.19	3	0.98
Sigmodon sp.	46	8.52	19	6.19
LAGOMORPHA				
Leporidae	46	8.52	16	5.21
UNIDENTIFIED	26	4.81	3	0.98

**Livestock category**	**12**	**2.22**	**0**	**0**
ARTIODACTYLA				
Bovidae				
Bos taurus	6	1.11	0	0
Capra hircus	4	0.74	0	0
Ovis aries	2	0.37	0	0
**Birds category**	**24**	**4.44**	**12**	**3.91**
GALLIFORMES				
Phasianidae				
Meleagris gallopavo	13	2.41	1	0.33
Unidentified	11	2.04	11	3.58
**Reptiles category**	**10**	**1.85**	**3**	**0.98**
**Invertebrates category**	**35**	**6.48**	**29**	**9.45**
COLEOPTERA	27	5.00	24	7.82
ORTHOPTERA	8	1.48	5	1.63
**Fruits category**	**370**	**68.52**	**259**	**84.36**
ERICALES				
Ericaceae				
Arctostaphylos pungens	159	29.44	91	29.64
PINALES				
Cupressaceae				
Juniperus deppeana	211	39.07	168	54.72

For gray foxes, we identified 17 different food items, belonging to 5 of the 6 trophic categories considered. The category with the highest frequency of occurrence value was fruits (FO_f = 84.36), followed by wild mammals (FO_{wm} = 28.66) and invertebrates (FO_i = 9.45; Table 1). We did not find any traces of livestock in gray fox feces. Within fruits category, *J. depeanna* was the most frequently consumed food item (FO_{Jd} = 54.72) followed by *A. pungens* (FO_{Ap} = 29.64). Within wild mammals category, rodents were the most frequent food item, represented by six species, among which the most consumed were of the genus *Peromyscus* (FO_P = 11.73), followed by species of the order Lagomorpha (FO_L = 5.21). Among the most frequent invertebrate food item were Coleoptera (FO_C = 7.82), while among birds, the most frequent prey items corresponded to unidentified species (FO_{NA} = 3.58; Table 1).

Trophic diversity was significantly higher (t = 7.03, df = 814.22, $P < 0.001$) for coyotes (H' = 2.33) than for gray foxes (H' = 1.80). While Horn index was R_0 = 0.934 (CI 95%; 0.898-0.969), which indicates high dietary overlap proportion, as well as very similar resource use spectra, between these two canid species.

Seasonal diet composition

Fisher's exact test showed significant differences in food items distribution across the trophic categories in species feces between seasons for coyotes ($P < 0.001$) and for gray foxes ($P < 0.001$). In the case of coyotes, food items in the wild mammals category were the most frequent remains in feces during winter (FO_{wm} = 60.32) and spring (FO_{wm} = 63.08), the first half of the year; while during the second half of the year were food items in the fruits category: summer (FO_f = 84.10) and autumn (FO_f = 66.67). Frequency of occurrence of birds (FO_b: 3.59–5.13) and invertebrates (FO_i: 4.17–7.18) was relatively constant throughout the year, and reptiles were present only in winter (FO_r = 1.59) and spring (FO_r = 4.10). Livestock were consumed in all seasons except for autumn, with low values between FO_d = 1.54 in summer and FO_d = 3.08 in spring (Figure 2, Table 2).

Figure 2. Seasonal occurrence of food items (expressed as proportion), by trophic categories, in the coyote and gray fox diets in the buffer zone of the La Michilía Biosphere Reserve, Durango, Mexico.

Table 2. Seasonal number of samples (*N*) and trophic diversity (*H'*) of the coyote and gray fox, as well as seasonal number of occurrences (n_i) and frequency of occurrence (*FO%*) of

food items by trophic category, in both canids diets, in the buffer zone of La Michilia Biosphere Reserve (MBR), Durango, Mexico.

	**Coyote**									
	**Winter**		**Spring**		**Summer**		**Autumn**		**Winter**	
	N **=126**		***N*** **= 195**		***N*** **= 195**		***N*** **= 24**		***N*** **= 87**	
	H' **= 2.16**		***H'*** **= 2.30**		***H'*** **= 1.47**		***H'*** **= 1.57**		***H'*** **= 1.88**	
**Food item and trophic category**	n_i	***FO%***	n_i	***FO%***	n_i	***FO%***	n_i	***FO%***	n_i	***FO%***
**Wild mammals category**	**76**	**60.32**	**123**	**63.08**	**50**	**25.64**	**9**	**37.50**	**39**	**44.83**
Mephitidae	6	4.76	3	1.54	3	1.54	0	0.00	1	1.15
N. narica	1	0.79	0	0.00	0	0.00	0	0.00	2	2.30
P. lotor	1	0.79	0	0.00	0	0.00	0	0.00	0	0.00
O. virginianus	4	3.17	11	5.64	2	1.03	0	0.00	0	0.00
P. tajacu	2	2.38	6	3.08	3	1.54	0	0.00	0	0.00
S. scrofa	4	3.17	3	1.54	2	1.03	0	0.00	0	0.00
S. nayaritensis	3	2.38	4	2.05	3	1.54	0	0.00	2	2.30
O. variegatus	1	0.79	2	1.03	2	1.03	2	8.33	1	1.15
T. umbrinus	0	0.00	5	2.56	2	1.03	0	0.00	2	2.30
H. irroratus	2	1.59	0	0.00	3	1.54	0	0.00	0	0.00
Peromyscus sp.	22	17.46	27	13.85	7	3.59	2	8.33	16	18.39
Reithrodontomys sp.	0	0.00	1	0.51	0	0.00	0	0.00	0	0.00
Sigmodon sp.	10	7.94	29	14.87	6	3.08	1	4.17	7	8.05
Leporidae	13	10.32	19	9.74	12	6.15	3	12.50	7	8.05
Unidentified	7	5.56	13	6.67	5	2.56	1	4.17	1	1.15
**Livestock category**	**3**	**2.38**	**6**	**3.08**	**3**	**1.54**	**0**	**0.00**	**0**	**0.00**
B. taurus	2	1.59	3	1.54	1	0.51	0	0.00	0	0.00
C. hircus	0	0.00	2	1.03	2	1.03	0	0.00	0	0.00
O. aries	1	0.79	1	0.51	0	0.00	0	0.00	0	0.00
**Birds category**	**6**	**4.76**	**10**	**5.13**	**7**	**3.59**	**1**	**4.17**	**5**	**5.75**
M. gallopavo	3	2.38	7	3.59	3	1.54	0	0.00	1	1.15
Unidentified	3	2.38	3	1.54	4	2.05	1	4.17	4	4.60
**Reptiles category**	**2**	**1.59**	**8**	**4.10**	**0**	**0.00**	**0**	**0.00**	**2**	**2.30**
**Invertebrates category**	**7**	**5.56**	**13**	**6.67**	**14**	**7.18**	**1**	**4.17**	**0**	**0.00**
Scarabaeidae	3	2.38	12	6.15	11	5.64	1	4.17	2	2.30
Orthoptera	4	3.17	1	0.51	3	1.54	0	0.00	0	0.00
**Fruits category**	**75**	**59.52**	**115**	**58.97**	**164**	**84.10**	**16**	**66.67**	**66**	**75.86**
A. pungens	61	48.41	87	44.62	10	5.13	1	4.17	35	40.23
J. deppeana	14	11.11	28	14.36	154	78.97	15	62.50	31	35.63

Gray foxes showed a different pattern of consumption than coyotes, since the most frequent trophic category in all seasons was fruits ($FO_f \geq 75.86$), followed by wild mammals,

which were most frequent in winter (FO_{wm} = 44.83) and spring (FO_{wm} = 36.96). During the summer and autumn, fruits category frequency increased, reaching a maximum value of FO_f = 92.00, and frequency of occurrence of wild mammals decreased (Figure 2, Table 2). The highest frequency of occurrence value of invertebrates was during the summer (FO_i = 23.08), and no invertebrates were found on its feces in autumn. On the other hand, birds *FO* values were relatively constant (3.85-5.75), except in autumn when no bird remains were found in the feces. Reptiles were present only in winter (FO_r = 2.03) and spring (FO_r = 1.09) seasons (Figure 2, Table 2).

The coyote's trophic diversity values were highest in winter and spring (*H'* = 2.34 and *H'* = 2.40, respectively), which did not differ significantly from each other (*t* = -0.55, *df* = 325.26, *P* = 0.58), and the minimum values were in summer (*H'* = 1.61) and autumn (*H'* = 1.57), which also did not differ significantly (*t* = 0.16, *df* = 39.62, *P* = 0.87). For this species, the remaining pairwise comparisons between seasons shows significant differences using Hutchenson's *t* tests (Table 3). In the case of gray foxes, like coyotes, the highest trophic diversity values occurred during winter (*H'* = 1.97) and spring (*H'* = 1.83), which did not statistically differ from each other (*t* = 1.02, *df* = 232.93, *P* = 0.31), while the lowest value occurred during the autumn (*H'* = 0.42). The rest of the comparisons between seasons showed significant differences (Table 3). When comparing the seasonal trophic diversity values between species, this parameter differed significantly during all seasons except for summer, when there was no evidence of statistically significant differences (*t* = 1.27, *df* = 267.16, *P* < 0.205; Table 3).

Table 3. Seasonal trophic diversity of coyotes and gray foxes, and significance values of Hutcheson's *t* test between seasons, in the buffer zone of La Michilia Biosphere Reserve (MBR), Durango, México.

			**Coyote**				**Gray fox**		
			**Winter**	**Spring**	**Summer**	**Autumn**	**Winter**	**Spring**	**Sum**
			H' = 2.16	H' = 2.30	H' = 1.47	H' = 1.57	H' = 1.88	H' = 1.77	H' =
**Coyote**	**Winter**	H' = 2.16	----						
	**Spring**	H' = 2.30	NS	----					
	**Summer**	H' = 1.47	***	***	----				
	**Autumn**	H' = 1.57	**	***	NS	----			
**Gray fox**	**Winter**	H' = 1.88	**	***	**	NS	----		
	**Spring**	H' = 1.77	***	***	NS	NS	NS	----	
	**Summer**	H' = 1.36	***	***	NS	NS	***	**	---
	**Autumn**	H' = 0.36	***	***	***	***	***	***	**

Minimum number of samples between comparisons was 74, degrees of freedom varied between 37.71–407.10. Significance values are indicated as follows: $P \leq 0.001$ "***", $P \leq 0.01$ "**", $P \leq 0.05$ "*", and $P > 0.05$ "NS".

Trophic niche overlap between coyotes and gray foxes through seasons sampled was high (≥ 82.10%). The highest overlap proportion value occurred in winter with R_0 = 0.905 (IC 95%; 0.823-0.986) and spring with R_0 = 0.945 (IC 95%; 0.866-0.998), then decreasing in summer with R_0= 0.870 (IC 95%; 0.821-0.919) and showing the lowest overlap in autumn with R_0 = 0.821 (IC 95%; 0.673-0.968).

Discussion

In the study area we found that fruits were the most frequently consumed food item year-round in coyote's diet, as has been reported for this species in coniferous forests habitats in Oregon and Maine [54]–[55] and in some desert, coastal, tropical deciduous forest, and urban environments [20], [56]–[60]. But, this results contrast with previous works in the MBR [21], [35], although former work represents a partial approximation of the diet throughout the year

during the summer and spring, and with works in other parts of coyote's distribution which report wild mammals as the most consumed trophic category year-round in temperate forest of Mexico [61]–[63], the United States of America [12], [64]–[70], and Canada [71]–[73]. However, despite the above, changes in the frequency of occurrence of the different trophic categories through the seasons coincide with data reported for this species for which the highest consumption of wild mammals occurred during the first half of the year, in winter and spring, and the main consumption of fruits occurred in the second part of the year, in summer and autumn [35], [60], [64], [70], [74]–[78]. Our results support that the coyote behaved as opportunistic feeder with general diet [64], [79]. This species is characterized by its adaptability to different habitat conditions, which is reflected in its diet, in such a way that coyotes took advantage of the seasonal availability of mammals and fruits. As was reported in the study area [35], we found that the highest consumption of wild mammals occurred in winter: mainly rodents, lagomorphs, and artiodactyls, which coincides with data reported for this species, since rodents of the genus *Peromyscus* and *Sigmodon* as well as lagomorphs (*L. californicus* and *S. audubonii*) were the elements that accounted for the highest proportion of consumption [10], [12], [21], [35], [61]. This species has also been reported to consume larger species, such as white-tailed deer (*O. virginianus*), which in the case of the present study had high *FO* values, below rodents and lagomorphs, but which may present higher importance values in other distribution areas [62], [64], [70], [72], [80]–[84]. These high values of mammal consumption during the first half of the year can be explained by the high demand for high-quality foods, since these periods coincide with the breeding season (1 January–15 March) and gestation (16 March–30 April [85]), such that foraging activity increases markedly in the study area to increase reproductive success [86]–[87]. On the other hand, in summer and autumn, fruit frequency was higher; the most consumed fruits were from cedar (*J. deppeana*), a species which has high availability during this season,

July-November [35], while the main consumption of *A. pungens* was during the winter and spring, which coincides with the low-water period in this area, just when the fruits of this shrub mature and fall to the ground; thus, coyotes consume the fruit and help disperse the seeds of this species which has an important role in this type of ecosystem, especially in restoring soils and retaining moisture at the beginning of secondary ecological succession [88]. Although our work shows that the rest of the food categories were complementary elements in the coyote diet, their importance and frequency of consumption vary depending on the habitat type where this canid resides, since in desert regions there is a higher consumption of invertebrates and reptiles [89]–[90] and in anthropized environments, an important consumption of livestock ($FO \geq 25\%$), including cattle, poultry, and domestic cats has been reported [91]–[94].

Overall feeding habits of the gray fox that we describe in this work, in which the main trophic category was fruits followed by wild mammals, coincides with the feeding pattern reported in similar biomes in North America [17], [66], [95]–[96], in central Mexico [97], in Guatemala [98], and in some areas of moist tropical forest in Mexico [20], [99] and Belize [100]. The gray foxes' most consumed trophic category throughout the year was fruits. However, the frequency of consumption of this and the rest of the categories differed significantly among seasons. During winter there was higher consumption of wild mammals, as has been reported in the central and eastern USA, where leporids and rodents are the gray fox's main prey [15], [101]. The lowest consumption of fruits occurred in the winter and increased gradually through the autumn, when it represented more than 90% of the diet. During autumn, foxes consumed almost exclusively cedar (*J. depeanna*) fruits. Invertebrates was also highly consumed by gray foxes, which have been reported to be mainly consumed in the summer [15], [16], [20], [102]–[104], the wettest season, as was the case in our study.

The variation in the frequency of consumption in the trophic categories that make up the diets of these two canids shows their ability to adapt to different habitat conditions. In the case of the coyote, it has been reported that this species responds to changes in resource availability by modifying their preferences when an important food source becomes less abundant [17], [35], [105]–[106]. This also appears to occur with gray foxes, since they have also demonstrated their adaptability to changes in the availability of food resources, whether due to stochastic events, temporal variation, or differences in the habitat types they occupy [15], [17], [20], [21], [99].

As expected, yearly trophic diversity of the coyote (*H'* = 2.33) was higher than that of the gray fox (*H'* = 1.80). Seasonally, this parameter differed between the two species in all seasons except for the summer, when the diversity of the dietary elements of the two species was more similar (82.15%). Coyotes had its highest trophic diversity in spring (*H'* = 2.40), and this was higher than that of the gray foxes (*H'* = 1.97). During this season, the coyote consumed three different species of livestock (*B. taurus*, *O. aries* and *C. hircus*) and three species of large wild mammals (*O. virginianus*, *P. tajacu* and the exotic *S. scrofa*), which the gray foxes did not consume at any time during the year. This is consistent with the prediction that sympatric carnivore species will partition prey species according to their body size [107], which may reflect the different energy requirements associated with size [12]. This has been previously reported in the coyote with respect to the gray fox [10], [21] and to other species of foxes in the Americas, such as the San Joaquín kit fox (*Vulpes macrotis mutica* [108]-[109]), the swift fox (*Vulpes velox* [12]), and the red fox (*Vulpes vulpes* [110]–[111]).

Overall (93.4%) and seasonally (82.1–94.5%) trophic niche overlap between coyotes and gray foxes in the study area was high, since both species consumed many of the same food items that were available in the MBR; this suggests that there could be exploitative competition. Our

results coincided with the only previous study investigating the similarity of the diets of these two sympatric canids in the study area, in which it was reported high trophic overlap, with a Pianka index value of *O* = 0.832 [21]. Given that the abundance of potential prey should be similar for both predator species, some of the differences detected in the use of prey could reflect differences in the feeding ecology of these two species [21], so competitive interactions over food resources are mediated by distinct foraging patterns that result in a differentiation of the consumption of some of the elements of the diets of these species. This resource partitioning pattern by ecologically similar species is a niche segregation strategy that facilitates these species' coexistence [5], [112]. However, it should also be pointed out that this difference in consumption patterns could also be due to differential use of the habitat (e. g. spatial segregation), which has been shown to be important in reducing competitive interactions between coyotes and gray foxes [16], [66].

Both canid species showed opportunistic food habits, although the trophic diversity of the coyote was higher than that of the gray fox, mainly because coyote incorporated larger prey into its diet than gray fox did not. However, the two species consumed quite similar range of food elements, that varied in proportion and frequency among seasons, so they showed high trophic niche overlap, and therefore high potential for exploitative competition in the study area.

Acknowledgements

We thank the authorities of Ejido San Juan de Michis and Anexo La Peña, Durango, Mexico. To M. Villa and P. Villa for the permits granted to carry out this work. To INECOL A. C. for allowing us to use the installations of "Piedra Herrada" Biological Station and to J. Medina and A. Guerra from that institution for their support. We are grateful to E. Chacón for logistical

support and to D. Carreón for the participation in this project. The Laboratorio de Ecología y Conservación de Fauna Silvestre of the UAM-X, VISILMEX A. C. and the Centro de Investigación en Biodiversidad y Conservación of the UAEM provided infrastructure, supplies, and all-terrain vehicles. This article was the result of the dissertation work of the first author to obtain a doctoral degree in the Doctorado en Ciencias Biológicas y de la Salud at the Universidad Autónoma Metropolitana.

References

1. Carvalho JC, Gomes P. Feeding resource partitioning among four sympatric carnivores in the Peneda-Gerês National Park (Portugal). J Zool. 2004;263(3):275–283.

2. Taper ML, Marquet PA. How do species really divide resources? Am Nat. 1996;147(6):1072–1086.

3. Lanszki J, Körmendi S, Hancz C, Zalewski A. Feeding habits and trophic niche overlap in a Carnivora community of Hungary. Acta Theriol. 1999;44(4):429–442.

4. Hardin G. The competitive exclusion principle. Science. 1960;131(3409):1292–1297.

5. Schoener TW. Resource partitioning in ecological communities. Science. 1974;185(4145):27–39.

6. Schoener TW, Roughgarden J, Fenchel T. The body-size-prey-size hypothesis: a defense. Ecology. 1986;67(1):260–261.

7. Xia J, Wu F, Hu WZ, Fang JL, Yang XJ. The coexistence of seven sympatric fulvettas in Ailao Mountains, Ejia Town, Yunnan Province. Zool Res. 2015;36(1):18–28.

8. Krebs CJ. Ecological methodology. 2nd ed. California: Addison-Wesley Educational Publishers, Inc.; 1999.

9. Azevedo FC, Lester V, Gorsuch W, Lariviere S, Wirsing AJ, Murray DL. Dietary breadth and overlap among five sympatric prairie carnivores. J Zool. 2006;269(1):127–135.

10. Neale JC, Sacks BN. Food habits and space use of gray foxes in relation to sympatric coyotes and bobcats. Can J Zool. 2001;79(10):1794–1800.

11. du Preez B, Purdon J, Trethowan P, Macdonald DW, Loveridge AJ. Dietary niche differentiation facilitates coexistence of two large carnivores. J Zool. 2017;302(3):149–156.

12. Kitchen AM, Gese EM, Schauster ER. Resource partitioning between coyotes and swift foxes: space, time, and diet. Can J Zool. 1999;77(10):1645–1656.

13. Glen AS, Dickman CR. Complex interactions among mammalian carnivores in Australia, and their implications for wildlife management. Biol Rev. 2005;80(03):387–401.

14. Bekoff M. *Canis latrans*. Mamm Species. 1977;79:1–9.

15. Fritzell EK, Haroldson KJ. *Urocyon cinereoargenteus*. Mamm Species. 1982;189:1–8.

16. Fedriani JM, Fuller TK, Sauvajot RM, York EC. Competition and intraguild predation among three sympatric carnivores. Oecologia. 2000;125(2):258–270.

17. Cunningham SC, Kirkendall L, Ballard W. Gray fox and coyote abundance and diet responses after a wildfire in central Arizona. West N Am Nat. 2006;66(2):169–180.

18. Larson RN, Morin DJ, Wierzbowska IA, Crooks KR. Food habits of coyotes, gray foxes, and bobcats in a coastal Southern California urban landscape. West N Am Nat. 2015;75(3):339–347.

19. Smith JA, Thomas AC, Levi T, Wang Y, Wilmers CC. Human activity reduces niche partitioning among three widespread mesocarnivores. Oikos. 2018;127(6):890–901.

20. Guerrero S, Badii MH, Zalapa SS, Flores AE. Dieta y nicho de alimentación del coyote, zorra gris, mapache y jaguarundi en un bosque tropical caducifolio de la costa Sur del Estado de Jalisco, México. Acta Zool Mex. 2002;86:119–137.

21. Delibes M, Hernández L, Hiraldo F. Comparative food habits of three carnivores in western Sierra Madre, Mexico. Z Säugetierkd. 1989;54(2):107–110.

22. Case TJ, Gilpin ME. Interference competition and niche theory. Proc Natl Acad Sci USA. 1974;71(8):3073–3077.

23. Servín J, Chacón E. Gray fox. In: Ceballos G, editor. Mammals of México. Maryland: Johns Hopkins University Press; 2014. pp. 514–515.

24. Servín J, Bejarano A, Alonso-Pérez N, Chacón E. El tamaño del ámbito hogareño y el uso de hábitat de la zorra gris (*Urocyon cinereoargenteus*) en un bosque templado de Durango, México. Therya. 2014;5(1):257–269.

25. Löwenberg-Neto P. Neotropical region: a shapefile of Morrone's (2014) biogeographical regionalisation. Zootaxa. 2014;3802:300–300.

26. Cuervo-Robayo AP, Ureta C, Gómez-Albores MA, Meneses-Mosquera AK, Téllez-Valdés O, Martínez-Meyer E. One hundred years of climate change in Mexico. PLoS ONE. 2020;15(7):e0209808.

27. Halffter G. Las Reservas de la Biosfera en el estado de Durango: una nueva política de conservación y estudio de los recursos bióticos. In: Halffter G, editor. Reservas de la biosfera en el estado de Durango. Veracruz: Instituto de Ecología AC; 1978. pp. 17–43.

28. Gadsden H, Reyes-Castillo P. Caracteres del ambiente físico y biológico de la Reserva de la Biosfera “La Michilía”, Durango, México. Folia Entomol Mex. 1991;81:1–19.

29. García E. Modificaciones al sistema de clasificación climática de Köppen (para adaptarlo a las condiciones particulares de la República Mexicana). 2nd ed. Mexico City: Instituto de Geografía, Universidad Nacional Autónoma de México; 1988.

30. Instituto Nacional de Estadística y Geografía (INEGI). Anuario estadístico y geográfico de Durango 2017. Aguascalientes: Instituto Nacional de Estadística y Geografía; 2017.

31. Gonzalez-Elizondo S, Gonzalez-Elizondo M., Cortes-Ortiz A. Vegetación de la reserva de la biosfera La Michilía, Durango, México. Acta Bot Mex. 1993;22:1–104.

32. Murie OJ, Elbroch M. A field guide to animal tracks (Vol. 3). New York: Houghton Mifflin Harcourt; 2005.

33. Kays RW, Wilson DE. Mammals of North America. 2nd ed. New Jersey: Princeton University Press; 2009.

34. Aranda M. Manual para el rastreo de mamíferos silvestres de México. Mexico City: Comisión Nacional para el Conocimiento y Uso de la Biodiversidad; 2012.

35. Servín J, Huxley C. La dieta del coyote en un bosque de encino-pino de la Sierra Madre Occidental de Durango, México. Acta Zool Mex. 1991;44:1–26.

36. Monroy-Vilchis O, Rubio-Rodríguez R. Guía de identificación de mamíferos terrestres del Estado de México, a través del pelo de guardia. Mexico: Universidad Autónoma del Estado de México; 2003.

37. Chomko SA. Identification of North American rodent teeth. In: Gilbert BM, editor. Mammalian osteology. Columbia: Missouri Archaeological Society; 1980. pp. 72–99.

38. Hillson S. Teeth. 1st ed. Cambridge: Cambridge University Press; 2005.

39. Elbroch M. Animal skulls: a guide to North American species. Pensilvania: Stackpole Books; 2006.

40. Álvarez-Castañeda ST, Álvarez T, González-Ruiz N. Guía para la identificación de los mamíferos de México en campo y laboratorio. Mexico City: Centro de Investigaciones Biológicas del Noroeste SC, Asociación Mexicana de Mastozoología AC; 2015.

41. Hillson S. Mammal bones and teeth: an introductory guide to methods of identification. New York: Routledge; 2016.

42. Breuer T. Diet choice of large carnivores in northern Cameroon. Afr J Ecol. 2005;43(3):181–190.

43. Erlinge S. Food studies on captive otters *Lutra lutra* L. Oikos. 1968;19(2):259–270.

44. Kasper CB, Peters FB, Christoff AU, Freitas TR. Trophic relationships of sympatric small carnivores in fragmented landscapes of southern Brazil: Niche overlap and potential for competition. Mammalia. 2016;80(2):143–152.

45. Klare U, Kamler JF, Macdonald DW. A comparison and critique of different scat-analysis methods for determining carnivore diet. Mamm Rev. 2011;41(4):294–312.

46. Colwell RK. EstimateS, version 9.1: Statistical estimation of species richness and shared species from samples. 2013. Available at: http://purl.oclc.org/estimates.

47. McDonald JH. Handbook of biological statistics. 3rd ed. Maryland: Sparky House Publishing; 2014.

48. Magurran AE. Ecological diversity and its measurement. New Jersey: Princeton University Press; 1988.

49. Hutcheson K. A test for comparing diversities based on the Shannon formula. J Theor Biol. 1970;29(1):151–154.

50. Hammer Ø, Harper DAT, Ryan PD. PAST: Paleontological statistics software package for education and data analysis. Palaeontologia electronica. 2001;4(1):1–9.

51. Horn HS. Measurement of "overlap" in comparative ecological studies. Am Nat. 1966;100(914):419–424.

52. Chao A, Chiu CH. Bridging the variance and diversity decomposition approaches to beta diversity via similarity and differentiation measures. Methods Ecol Evol. 2016;7(8):919–928.

53. R Core Team. R: A language and environment for statistical computing. Vienna: R Foundation for Statistical Computing; 2020.

54. Harrison DJ, Harrison JA. Foods of adult Maine coyotes and their known-aged pups. J Wildl Manage. 1984;48(3):922–926.

55. Toweill DE, Anthony RG. Coyote foods in a coniferous forest in Oregon. J Wildl Manage. 1988;52(3):507–512.

56. Murie A. Coyote food habits on a southwestern cattle range. J Mammal. 1951;32(3):291–295.

57. McClure MF, Smith NS, Shaw WW. Diets of coyotes near the boundary of Saguaro National Monument and Tucson, Arizona. Southwest Nat. 1995;40(1):101–104.

58. Quinn T. Coyote (*Canis latrans*) food habits in three urban habitat types of western Washington. Northwest Sci. 1997;71(1):1–5.

59. Guerrero S, Badii MH, Zalapa SS, Arce JA. Variación espacio-temporal en la dieta del coyote en la costa norte de Jalisco, México. Acta Zool Mex. 2004;20(2):145–157.

60. Schrecengost JD, Kilgo JC, Mallard D, Ray HS, Miller KV. Seasonal food habits of the coyote in the South Carolina Coastal Plain. Southeast Nat. 2008;7(1):135–144.

61. Aranda M, Rivera NL, De Buen LL. Hábitos alimentarios del coyote (*Canis latrans*) en la Sierra del Ajusco, México. Acta Zool Mex (ns). 1995;65:89–99.

62. Cruz-Espinoza A, González GE, Santos-Moreno A. Dieta del coyote (*Canis latrans*) en Ixtepeji, Sierra Madre de Oaxaca, México. Naturaleza y Desarrollo. 2010;8(1):33–45.

63. Martínez-Vázquez J, González-Monroy RM, Díaz-Díaz D. Hábitos alimentarios del coyote en el Parque Nacional Pico de Orizaba. Therya. 2010;1(2):145–154.

64. MacCracken JG, Uresh DW. Coyote foods in the Black Hills, South Dakota. J Wildl Manage. 1984;48(4):1420–1423.

65. Theberge JB, Wedeles CH. Prey selection and habitat partitioning in sympatric coyote and red fox populations, southwest Yukon. Can J Zool. 1989;67(5):1285–1290.

66. Cypher BL. Food item use by three sympatric canids in southern Illinois. Transactions of the Illinois State Academy of Science. 1993;86(3):139–144.

67. Arjo WM, Pletscher DH, Ream RR. Dietary overlap between wolves and coyotes in northwestern Montana. J Mammal. 2002;83(3):754–766.

68. Crimmins SM, Edwards JW, Houben JM. *Canis latrans* (coyote) habitat use and feeding habits in central West Virginia. Northeast Nat. 2012;19(3):411–420.

69. Dowd JL, Gese EM. Seasonal variation of coyote diet in northwestern Wyoming: Implications for dietary overlap with Canada lynx? Northwest Sci. 2012 86(4):289–299.

70. Cherry MJ, Turner KL, Howze MB, Cohen BS, Conner LM, Warren RJ. Coyote diets in a longleaf pine ecosystem. Wildlife Biol. 2016;22(2):64–70.

71. Messier F, Barrette C, Huot J. Coyote predation on a white-tailed deer population in southern Quebec. Can J Zool. 1986;64(5):1134–1136.

72. Patterson BR, Benjamin LK, Messier F. Prey switching and feeding habits of eastern coyotes in relation to snowshoe hare and white-tailed deer densities. Can J Zool. 1998;76(10):1885–1897.

73. Moehrenschlager A, List R, Macdonald DW. Escaping intraguild predation: Mexican kit foxes survive while coyotes and golden eagles kill Canadian swift foxes. J Mammal. 2007;88(4):1029–1039.

74. Bowyer RT, McKenna SA, Shea ME. Seasonal changes in coyote food habits as determined by fecal analysis. Am Midl Nat. 1983;109(2):266–273.

75. Andelt WF, Kie JG, Knowlton FF, Cardwell K. Variation in coyote diets associated with season and successional changes in vegetation. J Wildl Manage. 1987;51(2):273–277.

76. Ortega JC. Coyote food habits in southeastern Arizona. Southwest Nat. 1987;32(1):152–155.

77. Brillhart DE, Kaufman DW. Spatial and seasonal variation in prey use by coyotes in north-central Kansas. Southwest Nat. 1995;40(2):160–166.

78. Hidalgo-Mihart MG, Cantu-Salazar L, López-González CA, Martínez-Meyer E, González-Romero A. Coyote (*Canis latrans*) food habits in a tropical deciduous forest of western Mexico. Am Midl Nat. 2001;146(1):210–216.

79. Nellis CH, Keith LB. Population dynamics of coyotes in central Alberta, 1964–68. J Wildl Manage. 1976;40(3):389–399.

80. Ozoga JJ, Harger EM. Winter activities and feeding habits of northern Michigan coyotes. J Wildl Manage. 1966;30(4):809–818.

81. Andelt WF. Behavioral ecology of coyotes in south Texas. Wildl Monogr. 1985;94:3–45.

82. Parker GR. The seasonal diet of coyotes, *Canis latrans*, in northern New Brunswick. Can Field Nat. 1986;100(1):74–77.

83. McVey JM, Cobb DT, Powell RA, Stoskopf MK, Bohling JH, Waits LP, et al. Diets of sympatric red wolves and coyotes in northeastern North Carolina. J Mammal. 2013;94(5):1141–1148.

84. Hinton JW, Ashley AK, Dellinger JA, Gittleman JL, Manen FT, Chamberlain MJ. Using diets of Canis breeding pairs to assess resource partitioning between sympatric red wolves and coyotes. J Mammal. 2017;98(2):475–488.

85. Smith GJ, Cary JR, Rongstad OJ. Sampling strategies for radio-tracking coyotes. Wildl Soc Bull. 1981;9(2):88–93.

86. Servín J, Sánchez-Cordero V, Gallina S. Daily travel distances of coyotes (*Canis latrans*) in a temperate forest of Durango, Mexico. J Mammal. 2003;84:547–552.

87. Servín J, Chacón E, List R. Coyote. In: Ceballos G, editor. Mammals of México. Maryland: Johns Hopkins University Press; 2014. pp. 510–511.

88. Márquez-Linares MA, Jurado-Ybarra E, González-Elizondo S. Algunos aspectos de la biología de la manzanita (*Arctostaphylos pungens* HBK) y su papel en el desplazamiento de bosques templados por chaparrales. Ciencia UANL. 2006;9(1):57–64.

89. Hernández L, Delibes M, Hiraldo F. Role of reptiles and arthropods in the diet of coyotes in extreme desert areas of northern Mexico. J Arid Environ. 1994;26(2):165–170.

90. Grajales-Tam KM, Rodríguez-Estrella R, Cancino-Hernández J. Dieta estacional del coyote *Canis latrans* durante el periodo 1996-1997 en el desierto de Vizcaíno, Baja California Sur, México. Acta Zool Mex. 2003;89:17–28.

91. Gipson PS. Food habits of coyotes in Arkansas. J Wildl Manage. 1974;38(4):848–853.

92. Fedriani JM, Fuller TK, Sauvajot RM. Does availability of anthropogenic food enhance densities of omnivorous mammals? An example with coyotes in southern California. Ecography. 2001;24(3):325–331.

93. Santana E, Armstrong J. Food habits and anthropogenic supplementation in coyote diets along an urban-rural gradient. Hum–Wildl Interact. 2017;11(2):156–166.

94. Larson RN, Brown JL, Karels T, Riley SPD. Effects of urbanization on resource use and individual specialization in coyotes (*Canis latrans*) in southern California. PLoS ONE. 2020;15(2):e0228881.

95. Hockman JG, Chapman JA. Comparative feeding habits of red foxes (*Vulpes vulpes*) and gray foxes (*Urocyon cinereoargenteus*) in Maryland. Am Midl Nat. 1983;110(2):276–285.

96. Harrison RL. A Comparison of gray fox ecology between residential and undeveloped rural landscapes. J Wildl Manage. 1997;61(1):112–122.

97. Gómez-Ortiz Y, Monroy-Vilchis O, Mendoza-Martínez GD. Feeding interactions in an assemblage of terrestrial carnivores in central Mexico. Zool Stud. 2015;54(1):1–8.

98. Viteri-Pasch M, Mármol-Kattán A. Dieta de la zorra gris (*Urocyon cinereoargenteus*) y su posible importancia en la dispersión de semillas de ciprés (*Juniperus comitana*) en Huehuetenango, Guatemala. Revista Mexicana de Mastozoología (Nueva Época). 2019;9(1):66–71.

99. Villalobos-Escalante A, Buenrostro-Silva A, Sánchez-de la Vega G. Dieta de la zorra gris *Urocyon cinereoargenteus* y su contribución a la dispersión de semillas en la costa de Oaxaca, México. Therya. 2014;5(1):355–363.

100. Novaro A, Walker R, Suarez M. Dry-season food habits of the gray fox (*Urocyon cinereoargenteus fraterculus*) in the Belizean Peten. Mammalia. 1995;59(1):19–24.

101. Hatfield DM. Winter food habits of foxes in Minnesota. J Mammal. 1939;20(2):202–206.

102. Wood JE. Food habits of furbearers of the Upland Post Oak Region in Texas. J Mammal. 1954;35(3):406–415.

103. Fritzell EK. Gray fox and island gray fox. In: Novak M, Baker JA, Obbard ME, Malloch B, editors. Wild furbearer management and conservation in North America. Toronto: Ontario Ministry of Natural Resources; 1987. pp. 408–420.

104. Castellanos G, García Peña N, List R. Ecología del cacomixtle (*Bassariscus astutus*) y la zorra gris (*Urocyon cineroargenteus*). In: Lot A, Cano-Santana Z, editors. La Reserva Ecológica del Pedregal de San Ángel. Mexico City: Universidad Nacional Autónoma de México; 2009. pp. 371–381.

105. Leopold BD, Krausman PR. Diets of 3 predators in Big Bend National Park, Texas. J Wildl Manage. 1986;50(2):290–295.

106. Poessel S, Mock E, Breck S. Coyote (*Canis latrans*) diet in an urban environment: Variation relative to pet conflicts, housing density, and season. Can J Zool. 2017;95(4):287–297.

107. Rosenzweig ML. Community structure in sympatric Carnivora. J Mammal. 1966;47(4):602–612.

108. White PJ, Ralls K, White CAV. Overlap in habitat and food use between coyotes and San Joaquin kit foxes. Southwest Nat. 1995;40(3):342–349.

109. Cypher BL, Spencer KA. Competitive interactions between coyotes and San Joaquin kit foxes. J Mammal. 1998;79(1):204–214.

110. Green JS, Flinders JT. Diets of sympatric red foxes and coyotes in southeastern Idaho. Great Basin Nat. 1981;41(2):251–254.

111. Major JT, Sherburne JA. Interspecific relationships of coyotes, bobcats, and red foxes in western Maine. J Wildl Manage. 1987;51(3):606–616.

112. MacArthur R, Levins R. The limiting similarity, convergence, and divergence of coexisting species. Am Nat. 1967;101(921):377–385.

Figure 1

Click here to access/download;Figure;Fig1.tif

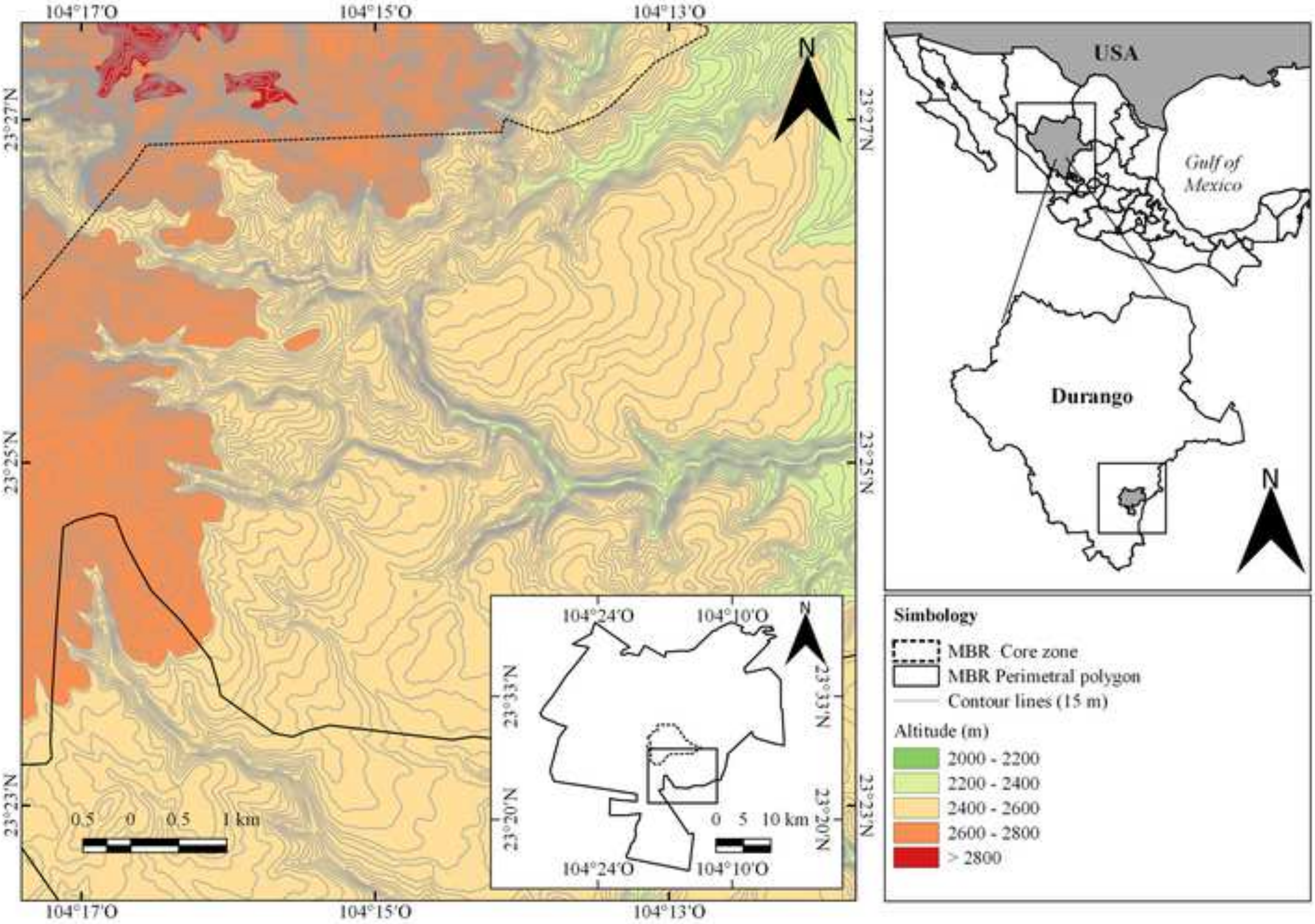

Figure 2

Click here to access/download;Figure;Fig2.tif

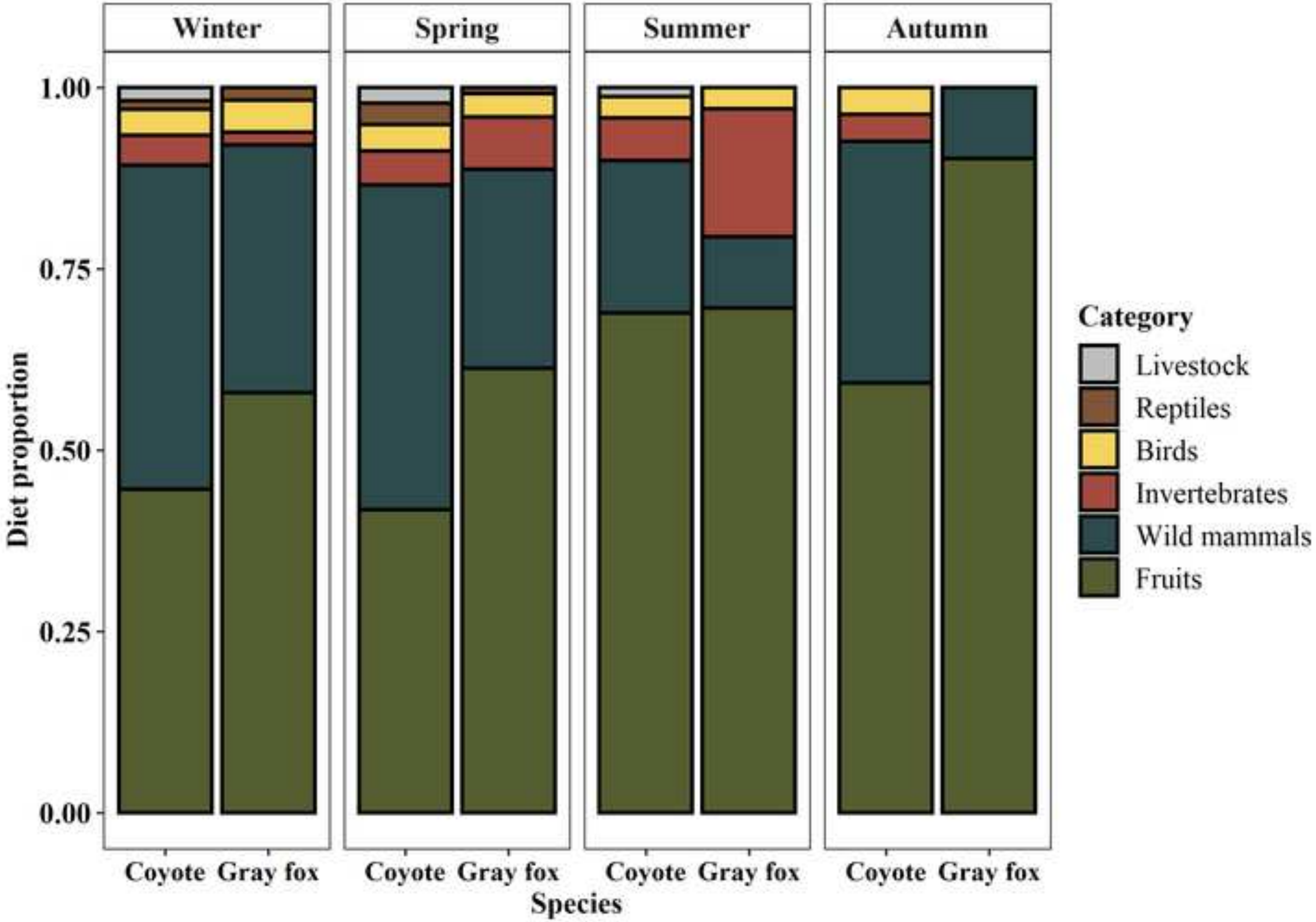

Supplement: Attachment — Submitted filename: PONE-D-21-19581.pdf [file pone.0260325.s003.pdf]
