# Supplementary material for: Trophic niche overlap between coyotes and gray foxes in a temperate forest in Durango, Mexico
Source: PLoS One. 2021 Dec 1;16(12):e0260325. doi: 10.1371/journal.pone.0260325 (PMC8635360; doi:10.1371/journal.pone.0260325)
Supplement: Attachment — Submitted filename: Response to Reviewers- PONE-S-21-24256.pdf [file pone.0260325.s004.pdf]

DETAILED DESCRIPTION ON HOW DO WE ATTEND ALL COMMENTS AND SUGGESTIONS DONE BY THE ASSOCIATE EDITOR AND BY TWO REVIEWERS

MANUSCRIPT PONE-S-21-24256 Trophic niche overlap between coyotes and gray foxes in a temperate forest in Durango, Mexico authored by César Ricardo Rodríguez-Luna, Jorge Servín, David Valenzuela-Galván (as corresponding author) and by Rurik List

Requests of the Associate Editor and/or by the journal

1. Please ensure that your manuscript meets PLOS ONE's style requirements, including those for file naming. The PLOS ONE style templates can be found at
https://journals.plos.org/plosone/s/file?id=wjVg/PLOSOne_formatting_sample_main_body.pdf and
https://journals.plos.org/plosone/s/file?id=ba62/PLOSOne_formatting_sample_title_authors_affiliations.pdf

R= We have done the required changes, and double checked that we do follow correctly PLOS ONE's style requirements in the newer corrected version we are now submitting.

2. Please include your full ethics statement in the 'Methods' section of your manuscript file. In your statement, please include the full name of the IRB or ethics committee who approved or waived your study, as well as whether or not you obtained informed written or verbal consent. If consent was waived for your study, please include this information in your statement as well.

R= Since our data is based on the collection and analysis of feces of the studied species, in México there is no need to obtain a permit for this. We do not collect and handle individuals of the studied species, therefore, we did not have to adhere to a particular ethic guideline for handling and studying animals.

In México, to be able to do research activities inside a Natural Protected Area, it is customary to inform the authorities of the NPA about the objectives and needs of the particular research; we did that before starting our research at La Michilia Biosphere Reserve. Since it is only needed to inform the authorities of the NPA, we only received verbal confirmation that they received our research protocol and that they do not had any issues in let in us to do our research, that provides useful information for the management of natural resources within the NPA.

3. In your Data Availability statement, you have not specified where the minimal data set underlying the results described in your manuscript can be found. PLOS defines a study's minimal data set as the underlying data used to reach the conclusions drawn in the manuscript and any additional data required to replicate the reported study findings in their entirety. All PLOS journals require that the minimal data set be made fully available. For more information about our data policy, please see http://journals.plos.org/plosone/s/data-availability.

"Upon re-submitting your revised manuscript, please upload your study's minimal underlying data set as either Supporting Information files or to a stable, public repository and include the relevant URLs, DOIs, or accession numbers within your revised cover letter. For a list of acceptable repositories, please see http://journals.plos.org/plosone/s/data-availability#loc-recommended-repositories. Any potentially identifying patient information must be fully anonymized.

Important: If there are ethical or legal restrictions to sharing your data publicly, please explain these restrictions in detail. Please see our guidelines for more information on what we consider unacceptable restrictions to publicly sharing data: http://journals.plos.org/plosone/s/data-availability#loc-unacceptable-data-access-restrictions. Note that it is not acceptable for the authors to be the sole named individuals responsible for ensuring data access.

We will update your Data Availability statement to reflect the information you provide in your cover letter

R= We have now uploaded a raw data base as supporting information that contains all needed data for anyone to perform the same analysis we did and present in our manuscript and to replicate the reported study findings in their entirety.

4. We note that Figure (1) in your submission contain map images which may be copyrighted. All PLOS content is published under the Creative Commons Attribution License (CC BY 4.0), which means that the manuscript, images, and Supporting Information files will be freely available online, and any third party is permitted to access, download, copy, distribute, and use these materials in any way, even commercially, with proper attribution. For these reasons, we cannot publish previously copyrighted maps or satellite images created using proprietary data, such as Google software (Google Maps, Street View, and Earth). For more information, see our copyright guidelines: http://journals.plos.org/plosone/s/licenses-and-copyright.

We require you to either (1) present written permission from the copyright holder to publish these figures specifically under the CC BY 4.0 license, or (2) remove the figures from your submission:

1. You may seek permission from the original copyright holder of Figure (1) to publish the content specifically under the CC BY 4.0 license.

We recommend that you contact the original copyright holder with the Content Permission Form (http://journals.plos.org/plosone/s/file?id=7c09/content-permission-form.pdf) and the following text:
"I request permission for the open-access journal PLOS ONE to publish XXX under the Creative Commons Attribution License (CCAL) CC BY 4.0 (http://creativecommons.org/licenses/by/4.0/). Please be aware that this license allows unrestricted use and distribution, even commercially, by third parties. Please reply and provide explicit written permission to publish XXX under a CC BY license and complete the attached form."

Please upload the completed Content Permission Form or other proof of granted permissions as an "Other" file with your submission.

In the figure caption of the copyrighted figure, please include the following text: “Reprinted from [ref] under a CC BY license, with permission from [name of publisher], original copyright [original copyright year].”

2. If you are unable to obtain permission from the original copyright holder to publish these figures under the CC BY 4.0 license or if the copyright holder’s requirements are incompatible with the CC BY 4.0 license, please either i) remove the figure or ii) supply a replacement figure that complies with the CC BY 4.0 license. Please check copyright information on all replacement figures and update the figure caption with source information. If applicable, please specify in the figure caption text when a figure is similar but not identical to the original image and is therefore for illustrative purposes only.
The following resources for replacing copyrighted map figures may be helpful:

USGS National Map Viewer (public domain): http://viewer.nationalmap.gov/viewer/
The Gateway to Astronaut Photography of Earth (public domain): http://eol.jsc.nasa.gov/sseop/clickmap/
Maps at the CIA (public domain): https://www.cia.gov/library/publications/the-world-factbook/index.html and https://www.cia.gov/library/publications/cia-maps-publications/index.html
NASA Earth Observatory (public domain): http://earthobservatory.nasa.gov/
Landsat: http://landsat.visibleearth.nasa.gov/
USGS EROS (Earth Resources Observatory and Science (EROS) Center) (public domain): http://eros.usgs.gov/#
Natural Earth (public domain): http://www.naturalearthdata.com/

R= figure 1 was created by CRRL (the first author) using shape files about topography and vegetation types and land cover produced by INEGI (Mexican National Institute of Geography and Statistics) and publicly available for free, for any user at the following link (https://www.inegi.org.mx/datos/?t=0150). Shape files were projected to produce figure 1 using QGIS software (v. 3.14.) that is free online to download at the following link (https://www.qgis.org/es/site/forusers/download.html). We stated now in our corrected version within the Methods section the source for this information and we stated now at the figure 1 caption that it was done in that way by CRRL and therefore we do not need to seek permission from anyone.

Reviewer's Comments to the Author

Reviewer #1:
This manuscript provides a simple, straightforward examination of dietary niche overlap between sympatric coyotes and gray foxes. As the authors point out, there is a lack of data examining the partitioning of these two ecologically similar and highly overlapping carnivores. I believe the authors’ research provides valuable information on the dietary niches of these two species.

R= we thank and appreciate reviewer’s considerations to our manuscript.

However, I also note a number of points that could be addressed to strengthen the manuscript.

1. The introduction could benefit from a bit more thoughtful expansion. The authors state that high trophic overlap for ecologically similar species is rare (line 45), but I might disagree. There are many sympatric generalist species, including carnivores (such as foxes, coyotes and bobcats), that can have high trophic overlap but coexist through mechanisms such as fine-scale spatial and/or temporal partitioning. In fact, the authors mention a study where coyotes and gray foxes can be found to have high dietary overlap (line 55). I believe it would strengthen the introduction to discuss the mechanisms which allow sympatric species with overlapping trophic niches to have stable coexistence. The authors mention that trophic niche segregation may allow stable coexistence (line 64), but what about circumstances (such as the findings in this manuscript) where trophic niche overlap is high but coexistence still appears to occur?

R= we have now added more information and a more thoughtful expansion on the issues mentioned by reviewer 1 to our introduction.

2. The transition from the broad introduction to the authors’ specific research seems a bit abrupt. Further discussion on how the reasoning and application of this research may be prudent.

R= We have added a bit more information about our reasoning of our research and why we consider that might provide insights on how the trophic niche segregation can explain partially the coexistence of sympatric ecologically similar species. Also, we modified the writing to solve the abrupt transition between the broad introduction and our specific research.

3. In the Sample Collection and Identification section, a few points may benefit from clarification which could improve future design replication:
a. How were transects allocated with respect to vegetation type? What was the length(s) of each transect?

R= We have provided now information about the number of transects, its length and location in relation to vegetation.

b. This is quite minor, but it feels inappropriate to say all feces were removed from the study area (line 99). Instead, “all feces were removed from the transects” might be more appropriate.

R= we accept and add this suggestion to our ms.

c. Were there any other sympatric carnivores that could be mis-identified as grey fox or coyote (e.g., Mephitidae species or other Vulpes species)? Especially if so, were all other scats cleared from transects during each survey?

R= In the studied area, there is only two wild canid species, but indeed, there are more sympatric carnivores, however, all produce quite distinctive feces and none can be easily confused with coyote or gray fox feces. In our original version of the manuscript, we stated that we identify all collected feces based on size, color, shape, length and maximum diameter and its comparison with the data for feces of different mammalian carnivore species published on field guides for different areas of the country. We discarded any collected feces that could be assigned to other species different of Coyote or Gray Fox. Asides, in the area, a previous study (Servin and Huxley, 1991) collected and measured different morphological data of a quite big sample of feces of Coyote and Gray Fox. We used the morphological data produced by them, to classify our collected feces as Coyote or Gray Fox feces, discarding all feces with maximum diameter falling in the overlapping area of the diameter distribution range of each species. We have added a bit more detail of this to our corrected version of the manuscript.

4. Although the authors mention sampling transects in each vegetation type, and that coyote food item consumption may vary by habitat type (line 276), vegetation type is not included in any analysis or discussion.

R= we have now changed our figure one to show the vegetation types and the distribution sampling transects in the studied area. Asides, in the text of the manuscript we briefly mention how many sampling transects were located on each vegetation type and we explain in the manuscript that fecal samples of both species were collected on all vegetation types and sampling was in accordance to the proportion of each habitat type. We added a couple of lines to our discussion stating that although feces were collected in all habitat types, not much inference about food resources consumed by habitat type can be derived from the location site of the feces sample, since an animal can consume resources at one place, and defecate several hours before at a different habitat type.

The authors find high dietary overlap, but do not discuss any other mechanisms which may facilitate stable coexistence. Examining the spatial overlap (site based or vegetation type based) of both species occurrences and dietary items may further illuminate the mechanisms which allow for sympatry of these two species. While this is briefly mentioned as a potential mechanism (lines 325 – 326), the authors likely have the necessary data to examine a spatial component as well (location and vegetation association of scat samples) to improve their findings.

R= We appreciate this reviewer's observation and we have now expanded our discussion to include information about any other mechanism that can facilitate stable coexistence.

5. The authors do a good job of thoroughly reviewing the findings in the discussion, but could strengthen their conclusions with greater connections back to the introduction. The authors do not relate back to their prediction in the introduction (lines 73 – 77), and I believe this would be important especially because the authors found high and not low trophic niche overlap. Due to these findings, further discussion on if/how stable coexistence may be occurring could be beneficial. The final conclusion in the discussion feels a bit lacking and leaves the reader wondering how these findings are applicable and where researchers/managers might benefit and move forward.

R= We also thank and appreciate this reviewer's observation, and we have considered and modified as suggested, our discussion.

6. The authors have generally written a nice manuscript, but it could be improved with some editing to correct grammar and improve sentence flow. A few examples are:
a. Lines 33 – 35: the first "difference" would read better as "differences"; "these different body sizes canid species" perhaps instead could be "these different sized canid species"
b. Line 47 and 59: what is "them" referencing?
c. Line 55: "its distribution the overlap of its diets" – what is "its" referencing?
d. Lines 61 – 65 and lines 73 - 77: these sentences are a bit hard to work through and could be improved with grammatical changes and/or being broken into two sentences.

R= we have done all suggested changes and also we reviewed our manuscript to edit and correct any grammar inconsistencies and tried to improve more our sentence flow.

<u>Reviewer #2</u>

The subject is interesting. However, it lacks many details of the methodology in the collection of samples. It is important to have information on the period with which the transects traveled, if in all the collections they traveled the same transects. This is due to the fact that generalist and opportunistic species such as the coyote and the gray fox can vary their diet in reduced time and space.

R= Similar request was done by reviewer 1. We have added all this detail to our corrected version of the manuscript, including information of sampling effort, sampling periods and if the same transects were travelled always.

On the other hand, the morphological characterization of the excrements would be convenient to strengthen it with some chemical or molecular technique; because its morphology is similar and the probability of error is high.

R= We have now provided more detail on the procedure we followed to minimize the potential misidentification of feces, and the conservative procedure implemented to assign with most confidence a feces sample to coyote or gray fox. The molecular or chemical methods suggested by the reviewer are indeed different procedures to assign to species a collected feces, however we already review all collected feces and we not have the possibility or the resources to implement those methods.

On the other hand, it is important to include complementary material with the characteristics of the hair of the identified species because several are similar.

R= We have now provided more detail on the procedure we followed to identify food items found on feces samples. Regarding its suggestion to include complementary material (e.g. microscopic slides), all the microscopic slides of guarding hairs we prepared with hairs obtained from the feces samples, are part of an ongoing undergraduate thesis work and therefore cannot be publicly shared yet. Those slides were compared to published microscopic images of guarding hairs of different mammal species that are included in several books, whose references were already cited in our original manuscript and still are in our corrected version of the ms.

We appreciate a lot the comments and observations done by reviewer 2, and we thank him to let us know who he is: Dr. Octavio Monroy-Vilchis, a researcher we followed and respect.
