# Supplementary material for: Trophic niche overlap between coyotes and gray foxes in a temperate forest in Durango, Mexico
Source: PLoS One. 2021 Dec 1;16(12):e0260325. doi: 10.1371/journal.pone.0260325 (PMC8635360; doi:10.1371/journal.pone.0260325)
Supplement: Attachment — Submitted filename: response-letter-plosone-nov2021.doc [file pone.0260325.s005.doc]

**DETAILED DESCRIPTION ON HOW DO WE ATTEND ALL COMMENTS AND SUGGESTIONS DONE BY THE ASSOCIATE EDITOR AND BY TWO REVIEWERS**

**Journal Requirements:**

Please review your reference list to ensure that it is complete and correct.

*R= We have done so; all cited references are in the reference list, that is complete and correct.*

If you have cited papers that have been retracted, please include the rationale for doing so in the manuscript text, or remove these references and replace them with relevant current references. Any changes to the reference list should be mentioned in the rebuttal letter that accompanies your revised manuscript. If you need to cite a retracted article, indicate the article’s retracted status in the References list and also include a citation and full reference for the retraction notice.

*R= we are not citing any retracted paper. We have not changed our previous reference list. We have done, only minor changes for some references (e.g. added page numbers for a reference that did not have that information).*

**Additional Editor Comments (if provided):**
**Some minor revisions:**

**L. 16: Resource partitioning, and especially dietary partitioning, is …..**

**L. 18: Mexico – without accent**

**L. 18: Better put ‘in Mexico’ after ‘widely distributed’**

**L. 20: HAVE not been**

**L. 40- understanding the ways in which species partition these resources**

**L. 46- IT is relatively common…**

**L. 61- ..THEIR coexistence..**

**L. 68- THESE canids**

**l. 70 – HAVE not been thoroughly studied**

**L. 77- a mechanism partly explaining**

**L. 127- We CHOSE**

**L. 128-131: ‘spp.’ must not be italicized, only the name of the genus goes in italics, e.g. Pinus spp.**

**L. 132- formal collection of FECAL samples**

**L. 134- however THEY all produce..**

**L. 135- careful to IDENTIFY feces**

**L. 138: IN the vicinity of the collection site (delete ‘DE’)**

**L. 145: Please rephrase (season considered and considered…)**

**L. 145: we assigned feces TO any..**

**L. 146- feces collected IN a particular season WERE representative…**

**L: 147: IN that period of the year.**

**L. 148-49: In THE laboratory….and washed THEM with water**

**L. 160: trough THEIR guarding hairs**

**L. 167: the overall and SEASONAL representation**

**L. 170: the percentage of feces that CONTAINS…and although IT DOES not necessarily…**

**L. 185: Mexico without accent**

**L. 190 : and that they DID NOT HAVE any issues in LETTING us do…**

**L. 194: FECAL samples**

**L. 201: we EXPLORED**

**L. 337: detected in coyote’s feces likely represent carrion…**

**L. 340: COINCIDE with the feeding patternS**

**L. 373-374- IN the area…..differences we found IN trophic diversity**

**L. 378: However, the relevance of each….**

**L. 379: IN the coyote’s than IN the gray fox diet**

**L. 379: Delete ‘and’ at the beginning of the sentence**

**L. 395: FECAL samples**

**L. 398-399: shouldn’t this be ‘ defecate several hours LATER..’? (not ‘before’)**

**L. 399: IN a different habitat type**

**L. 399- However, differences among habitats in the success…**

**L. 401: in accordance WITH our findings**

**L. 410 – FOR a better understanding**

**L. 411: can help minimize**

**L. 415: IN this Natural Protected Area**

*R= ALL 42 requested changes were done in the new version of the manuscript*

**Review Comments to the Author
Reviewer #1: The authors thoughtfully addressed the initial review comments. My only minor comment is that the manuscript requires a final proof-read to eliminate any typos and improve readability through correcting some grammar and sentence structure. A few examples from the Introduction, but not a thorough list, are below:
• Line 46: what is the subject of “is relatively common” ?
• Line 54 – 57: This sentence is a bit difficult to understand
• Line 57: “this kind” should be changed to “these kind” to match the plural of “interactions”
o Also on line 62: “this questions” should be changed to “these questions”
• Line 62: “choose” should be “chose”
• Line 63: there should not be a comma after “species”**

*R= ALL 6 requested changes were done in the new version of the manuscript.*

Asides, we have carefully reviewed the manuscript and corrected how we were using the square brackets for the references and some minor misspelling errors.
